# Levodopa Improves Cognitive Function and the Deficits of Structural Synaptic Plasticity in Hippocampus Induced by Global Cerebral Ischemia/Reperfusion Injury in Rats

**DOI:** 10.3389/fnins.2020.586321

**Published:** 2020-11-30

**Authors:** Wenzhu Wang, Xu Liu, Zhengyi Yang, Hui Shen, Lixu Liu, Yan Yu, Tong Zhang

**Affiliations:** ^1^Chinese Institute of Rehabilitation Science, China Rehabilitation Science Institute, Beijing, China; ^2^Beijing Key Laboratory of Neural Injury and Rehabilitation, China Rehabilitation Research Center, Beijing, China; ^3^School of Rehabilitation Medicine, Capital Medical University, Beijing, China; ^4^Institute of Automation, Chinese Academy of Sciences, Beijing, China; ^5^School of Biomedical Engineering, Tianjin Medical University, Tianjin, China; ^6^Beijing Bo'ai Hospital, China Rehabilitation Research Center, Beijing, China; ^7^Center of Neural Injury and Repair, Beijing Institute for Brain Disorders, Beijing, China

**Keywords:** levodopa, hippocampus, global cerebral ischemia reperfusion injury, structural synaptic plasticity, cognitive function

## Abstract

The cognitive impairment caused by cerebral ischemia/reperfusion is an unsolved problem in the field of international neural rehabilitation. Not only ameliorates the consciousness level of certain patients who suffered from ischemia-reperfusion injury and were comatose for a long time period after cerebral resuscitation treatment, but levodopa also improves the symptoms of neurological deficits in rats with global cerebral ischemia-reperfusion injury. However, Levodopa has not been widely used as a brain protection drug after cardiopulmonary resuscitation, because of its unclear repair mechanism. Levodopa was used to study the neuroplasticity in the hippocampus of global cerebral ischemia/reperfusion injury rat model, established by Pulsinelli's four-vessel occlusion method. Levodopa was injected intraperitoneally at 50 mg/kg/d for 7 consecutive days after 1st day of surgery. The modified neurological function score, Morris water maze, magnetic resonance imaging, Nissl and TH staining, electron microscopy and western blot were used in the present study. The results showed that levodopa improved the neurological function and learning and memory of rats after global cerebral ischemia/reperfusion injury, improved the integrity of white matter, and density of gray matter in the hippocampus, increased the number of synapses, reduced the delayed neuronal death, and increased the expression of synaptic plasticity-related proteins (BDNF, TrkB, PSD95, and Drebrin) in the hippocampus. In conclusion, levodopa can improve cognitive function after global cerebral ischemia/reperfusion injury by enhancing the synaptic plasticity in the hippocampus.

## Introduction

Sudden cardiac arrest is caused by organic diseases of the heart or other reasons that stop the ejection function of the heart, and the arterial pulsation, which in turn interrupts the blood circulation (Myerburg and Goldberger, 2017). This causes loss of consciousness and will subsequently result in serious hypoxia and ischemia of vital organs such as the heart, brain, and lungs. With the improvement of emergency treatment, the survival rate has increased up to 12%, but the rate of survival with good neurological function is <10% (Grasner et al., 2016; Writing Group et al., 2016). More than 45–70% of patients with global cerebral ischemia/reperfusion injury (GCI/R injury) have cognitive impairments, while serious cases among them are in persistent vegetative states (Fujioka et al., 1994; Lee et al., 2015), and milder cases exhibit memory dysfunctions such as the retrograde amnesia and amnesia (Benjamin et al., 2018). Cognitive impairment after the cardiopulmonary resuscitation, especially learning and memory dysfunction, is a major problem that needs to be urgently elucidated in the field of brain functional rehabilitation after cardiopulmonary resuscitation.

So far, the accepted treatment measures for the cerebral resuscitation are to minimize the time of early circulation interruption and cerebral hypoperfusion as well as cryotherapy and hyperbaric oxygen therapy (Geocadin et al., 2017; Ge et al., 2019). In 2017, the American Neurological Society issued clinical practice guidelines on reducing brain injury after cardiopulmonary resuscitation in which the main treatment option is still cryotherapy (Geocadin et al., 2017). Despite this, no brain protection drugs have so far proven to be effective (Geocadin et al., 2017). In recent years, NMDAR blockers were extensively studied for the repair of cognitive impairment caused by neuropsychiatric diseases such as stroke and Alzheimer's disease. However, due to their severe neurological side effects, most NMDAR blockers (such as MK801) ended in the failures related to the clinical studies (Zheng et al., 2017). At present, there are few brain protection drugs suitable for the early-stage treatment after clinical brain resuscitation. The development of brain protection drugs after cardiopulmonary resuscitation has thus become a hot spot within the international neuroscience research field.

The brain is the organ most sensitive to ischemia and hypoxia. Ischemia and hypoxia followed by cardiac arrest for more than 15 min usually lead to primary damage, aggravated structural damage, and functional damage to the brain tissue after the restoration of blood flow under certain conditions, that is, GCI/R injury (Schultz et al., 1996).

The central nervous system demonstrates selective vulnerability to the factors of ischemia and hypoxia. Even after a short period of ischemia, certain brain regions will be damaged. The hippocampus, which is closely related to cognition and memory, is one of the brain regions most vulnerable to ischemic damage (Cho et al., 2019).

Levodopa, as a precursor of dopamine, can penetrate the bloodbrain barrier, while dopamine itself cannot. Levodopa, therefore, is usually used to increase dopamine concentrations in the treatment of Parkinson's disease. Levodopa can improve learning and memory deficits in a mouse model of Alzheimer's disease (Ambree et al., 2009). Previous studies have shown that levodopa or piribedil can significantly improve the unconsciousness of patients with persistent coma after resuscitation (Lixu Liu and Shi, 2006). Moreover, levodopa can effectively improve the learning and memory of rats with cerebral ischemia/reperfusion injury (Wang et al., 2017) and reduce the death of hippocampal neurons caused by glucose and oxygen deprivation and reoxygenation injury (Wang et al., 2016). However, it is still unclear how levodopa repairs the cerebral ischemia/reperfusion injury. Currently, levodopa administration is mainly used as a conventional therapy for the supply of dopamine to the brain of patients with Parkinson's disease. But it is not yet used as a routine drug for the protection of the brain after CPR.

Although the hippocampus does not contain dopaminergic neurons and cannot produce dopamine, the fiber bundles can project dopamine from dopaminergic neurons and locus coeruleus (LC) in the ventral tegmental area (VTA) of the midbrain to the ventral and dorsal sides of the hippocampus (Fanselow and Dong, 2010; Kempadoo et al., 2016; McNamara and Dupret, 2017; Duszkiewicz et al., 2019). Dopamine, whether being projected on the ventral or dorsal side of the hippocampus, is involved in the regulation of learning and memory functions in the hippocampus (Tuesta and Zhang, 2014; Hagena et al., 2016; Kempadoo et al., 2016; Duszkiewicz et al., 2019). After dopamine innervation of the cerebral cortex neurons is lost, the length of the basal dendrites of the fifth-layer pyramidal neurons in the pre-frontal lobe will be shortened, and the density of dendritic spines in the pre-frontal and hippocampal CA1 neurons decreased (Wang and Deutch, 2008; Kasahara et al., 2015).

Our hypothesis is that the administration of levodopa can restore the dopamine content in the hippocampus, which improves the plasticity of neurons and repairs the learning and memory functions after GCI/R injury. In this study, we tried, from multiple perspectives, including behavioral science, magnetic resonance imaging, morphology of neurons and synapses in the hippocampus, and expression of molecules related to synaptic plasticity, to explore the repairing mechanism of levodopa to the learning and memory functions after GCI/R injury. We explored a new approach for the rehabilitation of brain function after cardiopulmonary resuscitation.

## Methods

### Experimental Animals

The animal experimental protocol (AEEI-2018-096) was approved by the ethics committee of Capital Medical University. Male Sprague-Dawley rats, weight 250–300 g, age 7–8 weeks, SPF (Specific pathogen Free) grade, were purchased from Beijing Weitong Lihua Experimental Animal Technology Co., Ltd. [license number: SCXK (Beijing) 2012-0001] and raised in the Animal Laboratory at the Chinese Academy of Rehabilitation Sciences. The random number table method was used to divide the experimental animals into three groups, i.e., the sham-operated group, the GCI/R injury model group (the model group), and the levodopa administration group.

### Preparation of GCI/R Injury Model

A modified Pulsinelli's four-vessel occlusion method was used to prepare the model. The bilateral vertebral arteries of the rats were electrocoagulated, and the bilateral carotid arteries were clamped with non-invasive vascular clamps for 20 min before the vascular clamps were released (Wang et al., 2019). The above method was used to prepare models in the model group and the levodopa administration group. In the sham-operated group, the bilateral vertebral arteries were not electrocoagulated and the common carotid arteries were not clamped, but other procedures were the same as the model group.

### Levodopa Intervention

Rats in the levodopa administration group were intraperitoneally injected with levodopa (50 mg/kg/d) for 7 consecutive days after the model was established. Levodopa (Sigma-Aldrich) and benzhydrazine hydrochloride (Sigma-Aldrich) were dissolved in normal saline at 4:1 (Levodopa is administered with benzhydrazine hydrochloride to prevent its peripheral conversion to DA.). Rats in the sham-operated group and model group were injected with an equal dose of normal saline.

### Modified Neurological Severity Scores

The modified neurological severity scores(mNSS) are made up of motor, sensory, reflex, and balance tests. Neurological function was graded on a scale of 0–18 (normal score, 0; maximal deficit score, 18). The mNSS test was performed at 3, 7, and 14 days after global cerebral ischemia/reperfusion. This study used the double-blind method for the scoring to achieve the final average value.

### Morris Water Maze

The water maze (Hong Kong Friends Honesty Life Sciences Co., Ltd., model XR-XM101) consists of a circular water tank with a diameter of 160 cm. The pool was divided into four quadrants. Each rat received three training trials per day for 5 consecutive days. The time to search for hidden platforms was 120 s. Once the rat found the submerged platform, it was allowed to stay on it for 10 s, and the time of absconding was recorded. On the 6th day, the platform was removed for space exploration experiment, and the rats swam freely for 120 s. The time of staying in the target quadrant and the frequency of rats crossing the platform were recorded.

### Magnetic Resonance Examination

#### MRI Scan

Magnetic resonance imaging was performed on rats in each group on 3, 7, and 14 days after GCI/R injury. The equipment was 7.0 T MRI scanner (PharmaScan, Bruker Biospin, Rheinstetten, Germany). T2WI and DTI of rat brain were acquired, and the specific series of parameters are as follows. T2: The sequence type was RARE Slice thickness: 0.3 mm, Gap: 0 mm, Slice: 90 layers, TR = 10,700 ms, TE = 36 ms, Flip Angle = 180°, the phase-encoding direction was from left to right, and the number of superimposed layers was four times. DTI: The sequence type was single-shot EPI (echo-planar imaging), Slice: 60 layers, TR = 15,000 s, TE = 22.4 ms, flip angle = 90°, b value 1 = 0, b value 2 = 1000 s/mm^2^, 30 gradient directions, the phase-encoding direction was from left to right, and the number of superimposed layers was one time.

#### Voxel-based Morphometry (VBM) Analysis

The VBM was used to analyze the magnetic resonance images of the rat brain structure. The function of Segment in the SPM12 official software based on the MATLAB9.3 platform was used to perform color unevenness correction, spatial standardization, and tissue segmentation as well as to divide the entire image into gray matter, white matter, cerebrospinal fluid, and non-cerebral voxels. After segmentation, the voxel size was 0.125 × 0.125 × 0.125 mm^3^. The segmented gray matter image modulated by the deformation field was used to compare the gray matter density between groups. A Gaussian kernel with a full width at the half maximum (FWHM) of 0.3 mm was used for spatial smoothing to increase the effectiveness of the parameters and to reduce the noise caused by registration errors. The two-sample *t*-test was used to compare different voxel groups, and a method based on the random field theory was used to correct for multiple comparisons. The threshold value was *P* = 0.05, and a cluster size of more than 200 was regarded as a statistically significant difference. ITK-SNAP3.8.0 and MIPAV8.0.2 were used to display the results of analysis.

In order to clearly display the anatomical position of the difference zone, we used the MIPAV8.0.2 software to carry out three-dimensional drawing of the difference zone in the hippocampus. First, the Marching Cube algorithm was used to extract the surface of the rat brain tissue, the surface of the difference-side hippocampus, and the surface of the difference zone. Then, the surface of the brain tissue and the surface of the hippocampus were set to be translucent to show the relative spatial position between the difference zone and the hippocampus.

#### Analysis of DTI Data

In the MRI laboratory, two experimental staffs collected diffusion tensor imaging (DTI) data, selected the largest area of the hippocampus in the rat coronal MRI image, and took bilateral hippocampus as the regions of interest. FA was calculated in the regions of interest in the hippocampus, and the average values of both sides were analyzed.

### Nissl and Tyrosine Hydroxylase (TH) Staining

A total of 3, 7, and 14 days after reperfusion, the perfusion fixation with paraformaldehyde was carried out for six rats in each group. Brains were taken out and placed in 4% paraformaldehyde solution in a 4°C refrigerator for 24 h. Dehydration and embedding were carried out according to the conventional method to make wax blocks of hippocampus tissues. A total of 3um paraffin sections were made and treated with Nessler staining. The sections were stained with aniline blue staining solution for 5–10 min. After being washed with distilled water, they were mounted transparently. At the same time, paraffin sections were immunostained with the anti-TH antibody (1:1,000, Wuhan Servicebio Technology Co., Ltd., China). The TissueGnostics tissue analysis system (TissueGnostics, Vienna, Austria) was used to scan the stained pathological sections.

For Nissl staining and immunostaining, serial sections from Bregma −2.2 to −4.2 mm were selected at intervals of every sixth section from each rat for quantification. The CA1 and CA2 areas of the hippocampus were examined at 20× magnification using Tissue FAXS Viewer software (TissueGnostics, Vienna, Austria). The number of survival neurons with intact membranes and nuclei in the hippocampal CA1 and CA2 regions were manually counted within three non-overlapping fields under the area 220 × 350 μm^2^. There were four rats in each group and three sections were chosen from each rat. The number of survival neurons was calculated as the average neuron number per field on each section.

For TH immunohistochemical stained sections, the average optical density (OD) was quantified by measuring intensity in hippocampus using Image J software. Data were analyzed from three sections of one sample, and there were four samples in each group.

### Synapse Ultrastructure

A total of 7 and 14 days after reperfusion, perfusion fixation was carried out for the rats in each group. Specimens of hippocampal CA1 region 1 mm^3^ in size were taken out quickly and placed in 2.5% glutaraldehyde for 2 h and washed with 0.1 mol/L PB buffer. The specimens were then fixed with 1% osmium acid solution for 2 h and washed, dehydrated with graded ethanol, and embedded with resin. Tissue sections were made and stained with uranyl acetate and lead citrate. Finally, under the HT7700 Transmission Electron Microscope (HT7700, Hitachi, Japan), ultrastructural pictures of neuronal synapses in the hippocampal CA1 region were collected, the number of synapses at 6,000× magnification, and the number of single synaptic vesicles at 20,000× magnification was counted. Statistical analysis was then conducted.

### Western Blot

After decapitation of the rat, the hippocampus tissue was quickly taken out, weighed, added with cell lysate, and ground. It was then thoroughly homogenized and centrifuged, and treated with protein electrophoresis. The extracted protein was separated using 10% SDS-PAGE, and then electro-transferred to a polyvinylidene fluoride membrane. Subsequently, at room temperature, the membrane was blocked in 5% skim milk powder dissolved in TBST for 1 h. It was then incubated with rabbitanti-caspase 3 antibody (1:1,000, Cell Signaling Technology), anti-bax antibody (1:1,000, Cell Signaling Technology), and anti-bcl2 antibody (1:1,000, Cell Signaling Technology) overnight at 4°C. The membrane was then incubated with IRDye-conjugated goat anti-rabbit antibody (Thermo Scientific, USA) for 2 h to develop color. The BIO-RAD confocal laser scanning imaging system (Bio-Rad Laboratories, Inc, USA) was used for scanning. The Image J software was used to obtain the gray value of the target band for statistical analysis.

### Statistical Analysis

SPSS22.0 software was used for the statistical analysis of the data in the present study. GraphPad prism7 software was used to make statistical graphs. The data are expressed as mean ± standard deviation, and we tested that whether the data meet normal distribution and homogeneity of variance. One-way analysis of variance (ANOVA) was used to compare the data between the three groups at the same time, repeated measures analysis of variance was used for the analysis of modified NSS scores and the escape latency in the water maze. If the variances were homogeneous, the LSD test was used as a *post-hoc* test. If the variances were not homogeneous, the Dunnett's T3 test was used as a *post-hoc* test. In this study, a significance level of 0.05 was adopted.

## Results

### Levodopa Reduced the Neurological Function Score of Rats After GCI/R Injury

**Figure 1:**
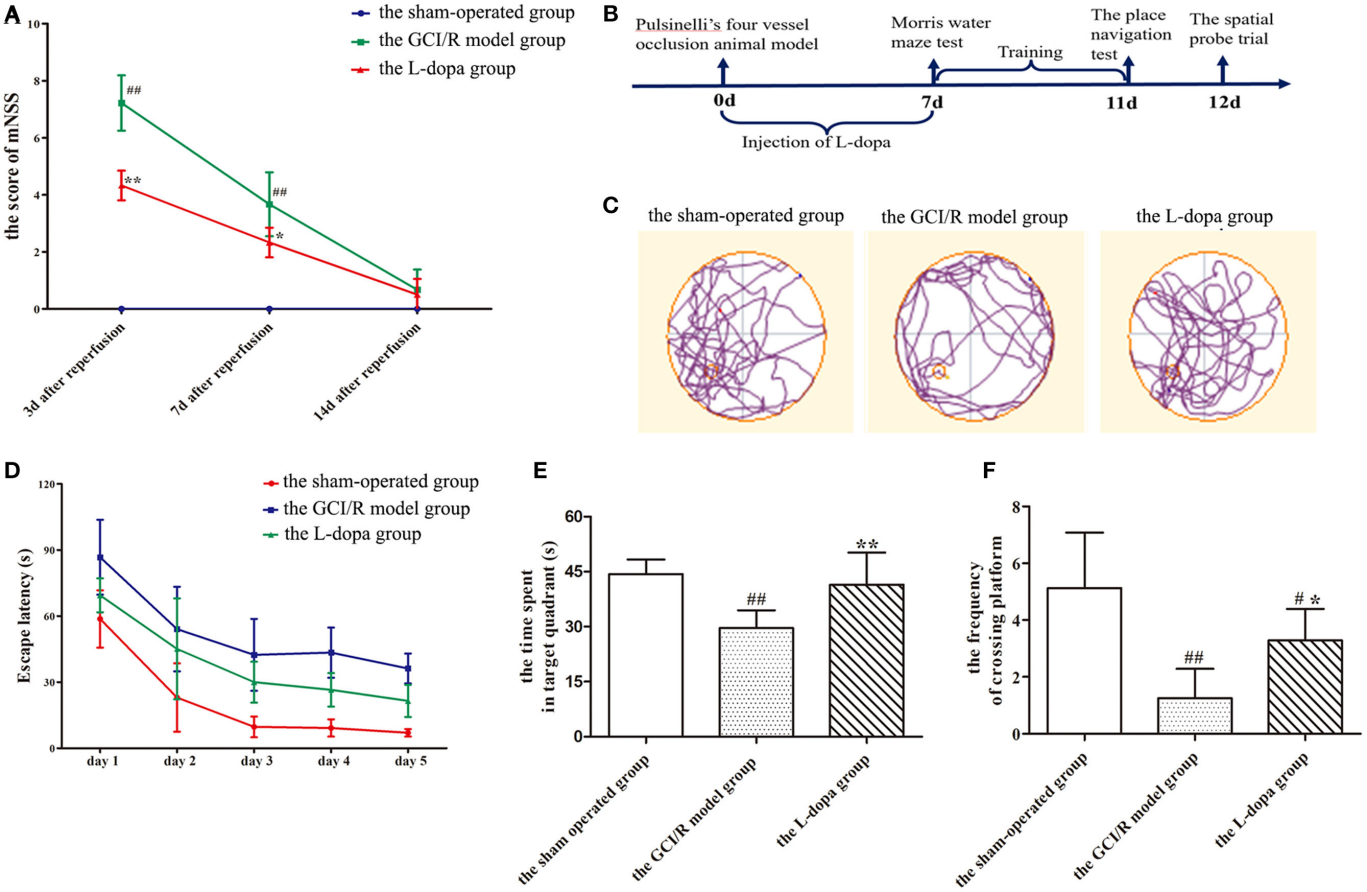
Levodopa improves cognitive impairment after GCI/R injury. **(A)** mNSS of the rats at 3, 7, and 14 days after GCI/R injury in each group. **(B)** Experimental timeline for Morris water maze. Administration of levodopa 7 consecutive days after GCI/R injury produces learning and memory recovery in rats as assessed in Morris water maze. Each rat received three training trials per day for 5 consecutive days. On the 11th day, the place navigation was carried out. Next day, the platform was removed for spatial probe trial. **(C)** Swimming paths of the rats in each group during the Morris water maze. **(D)** The escape latency during the training period of 5 consecutive days. **(E)** The time spent in target quadrant. **(F)** The frequency of crossing platform. Data are shown as means ± SD. ##*p* < 0.01 vs. the sham-operated group, **p < 0.01 vs. the GCI/R model group. #*p* < 0.05 vs. the sham-operated group, *p < 0.05 vs. the GCI/R model group.

At 3, 7, and 14 days after reperfusion, the mNSS scores of the sham-operated group, the GCI/R injury model group, and the levodopa administration group was recorded (Figure 1A). The scores were statistically compared using two-way analysis of variance. The variance was not homogeneous (*p* < 0.001), and there were differences between the three groups [*F*_(8, 57)_ = 45.366; *p* < 0.001]. The Dunnett's T3 test was used as a *post-hoc* test. The mNSS scores of the sham-operated group were all 0 points. The results showed that, compared with the mNSS scores at 7 and 14 days, the mNSS score at 3 days was the highest (*p* < 0.001), and the mNSS scores at 7 and 14 days continued to decline (Figure 1A). Compared with the sham-operated group and the levodopa administration group, the mNSS score of the GCI/R injury model group was the highest (*p* < 0.001), and the mNSS score of the drug administration group was lower than that of the model group (*p* < 0.001).

### Levodopa Improved the Cognitive Function of Rats With GCI/R Injury

In the test of place navigation, the data of 5 consecutive days of escape latency in the sham-operated group, the GCI/R injury model group and the levodopa administration group was analyzed by two-factor repeated measures ANOVA, and the results did not meet the test of sphericity (*p* = 0.001). After applying the Greenhouse-Geisser correction, it was found there were differences between groups at different time points [*F*_(14, 100)_ = 26.704, *p* < 0.001]. Multivariate analysis of variance showed that compared with the sham-operated group and the drug administration group, rats in the model group had the longest escape latency (*p* < 0.001), and rats in the drug administration group had shorter escape latency than the model group (*p* < 0.001), though it was still higher than the model group (*p* < 0.001) (Figure 1D).

In the space exploration experiment, the data of the time of rats spending in the target quadrant and the frequency of rats crossing the platform met the homogeneity of variance (*p* > 0.05) (Figure 1E). Single-factor analysis of variance was used for statistical analysis, and the LSD test was used as a *post-hoc* test. The results showed that there were statistical differences between the three groups during the time rats spent in the target quadrant and the frequency of rats crossing the platform [*F*_(2, 20)_ = 12.948, *p* < 0.001; *F*_(2, 20)_ = 14.379, *p* < 0.001]. The time of rats at the target quadrant and the frequency of rats crossing the platform in the model group were less than those in the sham-operated group (*p* < 0.001). The time of rats at the target quadrant and the frequency of rats crossing the platform in the levodopa administration group were more than those in the model group (*p* < 0.001) (Figure 1F). The swimming trajectory of rats in the space exploration test showed that rats in the model group appeared mainly to swim along the periphery of the pool and the time of rats spending in the target quadrant was less, while the swimming trajectories of rats in the sham-operated group and the drug administration group were very close within the target quadrant around the platform (Figure 1C).

### Levodopa Improved the Integrity of White Matter of Rat Hippocampus After GCI/R Injury

**Figure 2:**
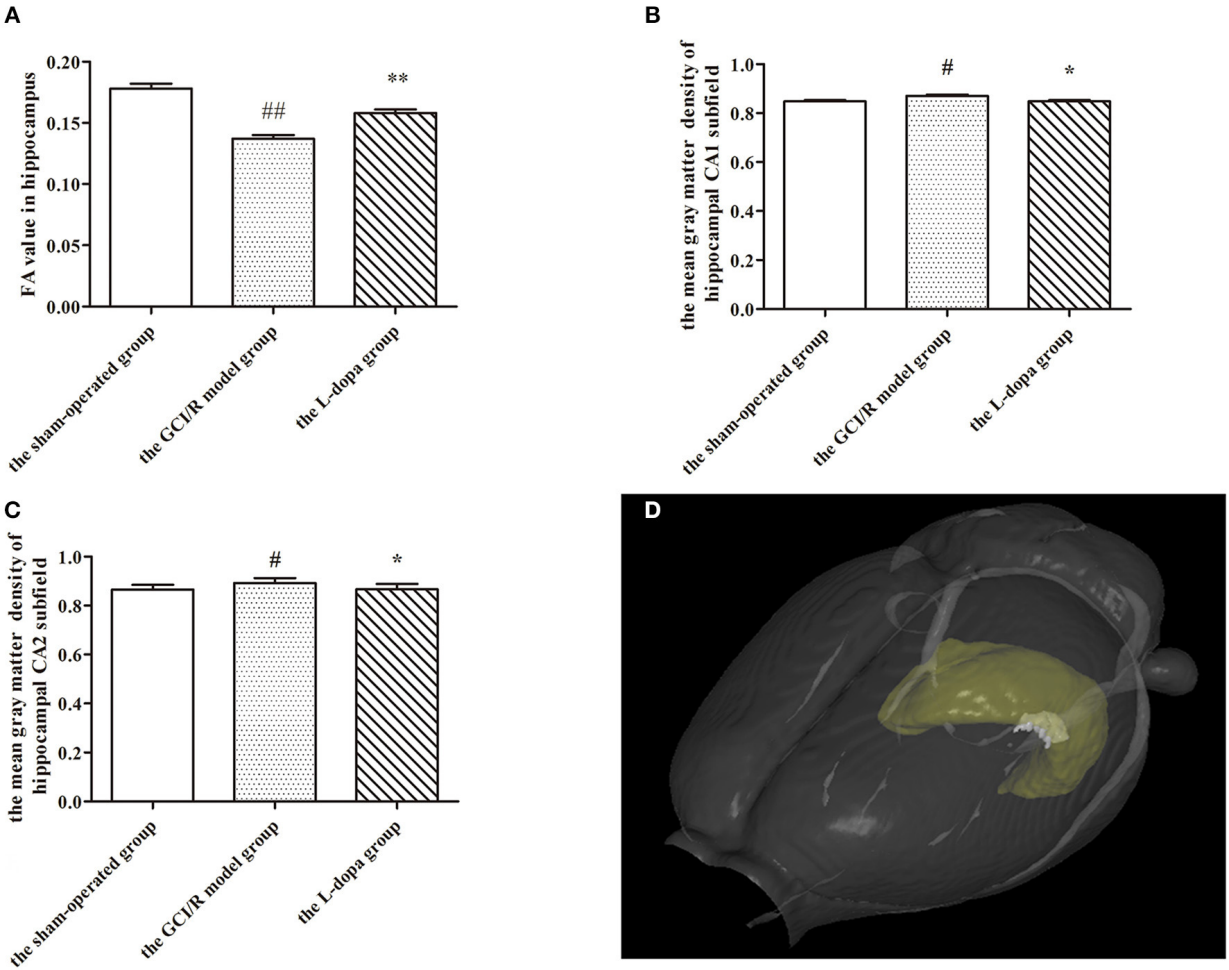
MRI imaging data. **(A)** Comparison FA value in hippocampus among groups. The mean gray matter density of hippocampal CA1 **(B)** and CA2 **(C)** subfield. **(D)** A three-dimensional (3D) rendering of regions with significant differences in gray matter density among groups. Data are shown as means ± SD. ##*p* < 0.01 vs. the sham-operated group, **p < 0.01 vs. the GCI/R model group. #*p* < 0.05 vs. the sham-operated group, *p < 0.05 vs. the GCI/R model group.

DTI can reflect the diffusion of water molecules and the integrity of white matter fiber bundles. In this study, MRI-DTI was used to detect the FA value of hippocampus in three groups at 3, 7, and 14 days after reperfusion injury. One-way analysis of variance was used to compare the FA values of rats in the sham-operated group, the model group, and the drug administration group at the same time. The results of the three groups at three time points showed homogeneity of variance (*p* > 0.05), and the LSD test was used as a *post-hoc* test. The FA values at different time points were different between the sham-operated group, model group, and drug administration group [*F*_(2, 16)_ = 30.383, *p* < 0.01; *F*_(2, 12)_ = 42.802, *p* < 0.01; *F*_(2, 17)_ = 9.791, *p* < 0.01], and the FA values of the drug administration group at three time points were higher than those of the model group (*p* < 0.01; *p* < 0.01; *p* < 0.05) (Figure 2A).

### Levodopa Improved the Gray Matter Density of Rat Hippocampus After GCI/R Injury

This study used voxel-based morphology to analyze the changes of each voxel in the MRI images of hippocampal CA1, CA2, CA3, and DG subregions on the third, seventh, and 14th days after reperfusion injury. One-way analysis of variance was used to determine the gray matter density of hippocampal CA1, CA2, and CA3 subregions in three groups. The gray matter densities of hippocampal CA1 and CA2 subregions in the model group were higher than those in the sham-operated group [*F*_(2, 15)_ = 6.849, *p* < 0.01; *F*_(2, 15)_ = 3.353, *p* < 0.05], but lower than those in the drug administration group (*p* < 0.01; *p* < 0.05) (Figures 2B,C). The gray matter densities of the CA1 and CA2 subregions in the drug administration group were not different from those in the sham-operated group. There were no differences in the gray matter densities of the hippocampal CA3 and DG subregions between the three groups [*F*_(2, 15)_ = 4.008, *p* > 0.05; *F*_(2, 15)_ = 1.900, *p* > 0.05]. However, we found out a different region of voxel 249 in the hippocampal CA3 subregion and detected that the average gray matter density of this region in the drug administration group was 1.02, in the control group it was 1.023, and in the model group it was 1.072 (Figure 2D). The results of the VBM study showed that the gray matter density of the hippocampus after levodopa administration was closer to that of the sham-operated group. The overall repair was significant in the hippocampal CA1 and CA2 subregions and the partial repair was significant in the hippocampal CA3 subregion.

### Levodopa Improved the Neuronal Morphology of Rat Hippocampus After GCI/R Injury

In the sham-operated group, pyramidal cells in the hippocampus were arranged neatly and densely, with clear nucleoli, abundant cytoplasm, and complete cell morphology and structure. There were numerous deep-colored Nissl bodies with large particles. Compared with the sham-operated group, the pyramidal cells in the hippocampus of rats in the model group were arranged disorderly, the number was reduced, the structure was indistinct, the neurons showed nuclear shrinkage, the Nissl bodies in the cytoplasm were light-colored with the reduced number, the neurons were vacuolated significantly, and some pyramidal cells indicated neuronal damage in the hippocampus. The neuronal morphology of the levodopa administration group was closer to that of the model group, the neurons were arranged more neatly, and the nuclear shrinkage of the neurons was reduced.

**Figure 3:**
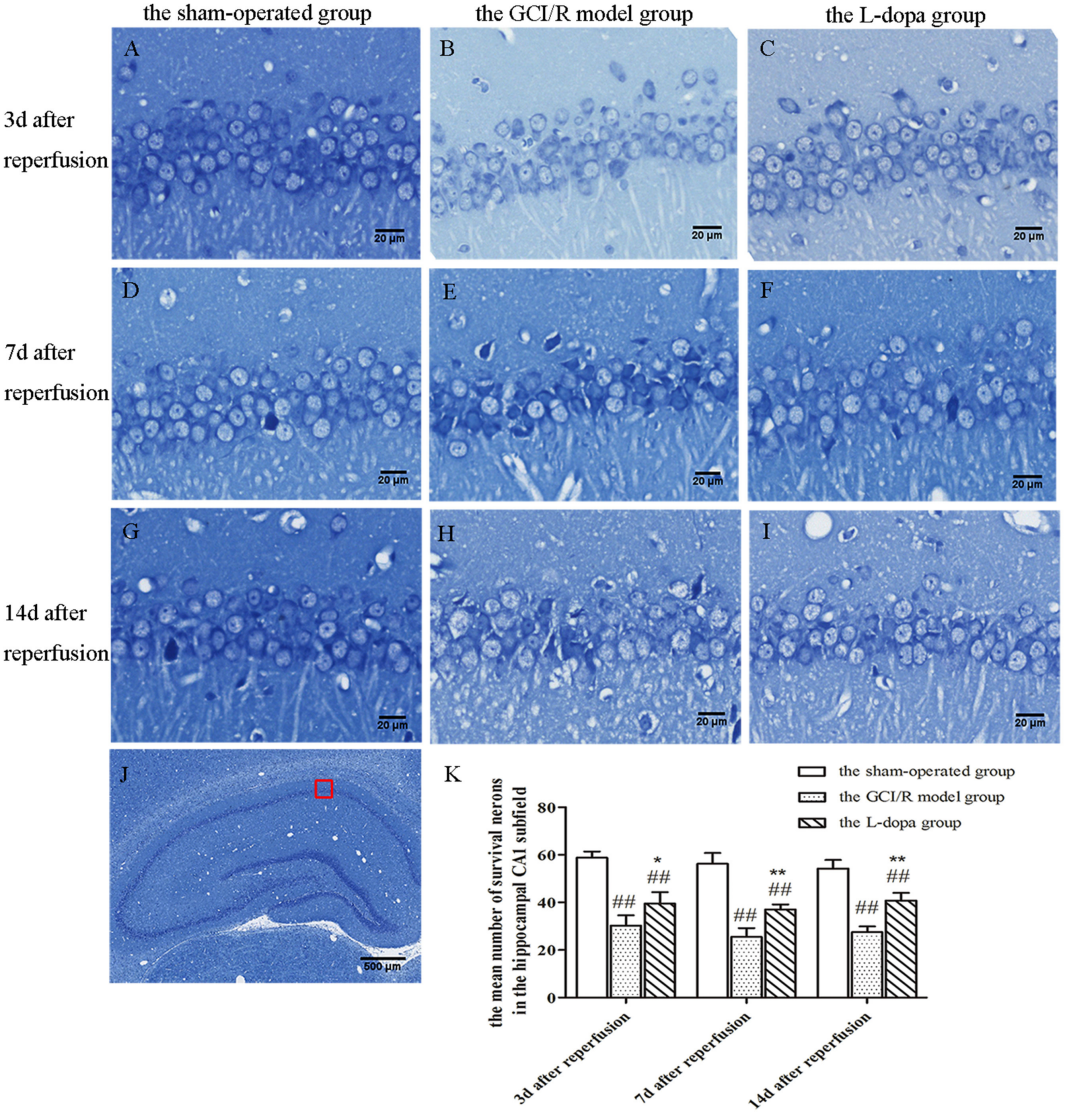
Nissl staining of hippocampal CA1 subfield. **(A–J)** Representative section for Nissl staining at 3, 7, and 14 days after GCI/R injury. Scale bar 500 μm for Magnification 4×, Scale bar 20 μm for Magnification 20×. **(K)** The mean number of survival neurons in the hippocampal CA1 at 3, 7, and 14 days after GCI/R injury. Data are shown as means ± SD. ##*p* < 0.01 vs. the sham-operated group, **p < 0.01 vs. the GCI/R model group. *p < 0.05 vs. the GCI/R model group.

The hippocampal CA1 region was selected under 20× objective lens for neuron counting. The sham-operated group, the model group, and levodopa administration group were counted at the same time, and the result was analyzed by single factor analysis of variance. The result showed that 3, 7, and 14 days after reperfusion, neuron counts in the hippocampus of the three groups all showed homogeneity of variance (*p* > 0.05), and the LSD test was used as a *post-hoc* test. There were statistical differences between the three groups [*F*_(2, 9)_ = 51.947, *p* < 0.001; *F*_(2, 9)_ = 75.078, *p* < 0.001; *F*_(2, 9)_ = 72.771, *p* < 0.001], the number of normal neurons in the global ischemia/reperfusion injury model group and the levodopa administration group were significantly smaller than that in the sham-operated group (*p* < 0.01). Compared with the model group, the number of neurons in the drug administration group increased significantly (*p* < 0.01) (Figure 3).

**Figure 4:**
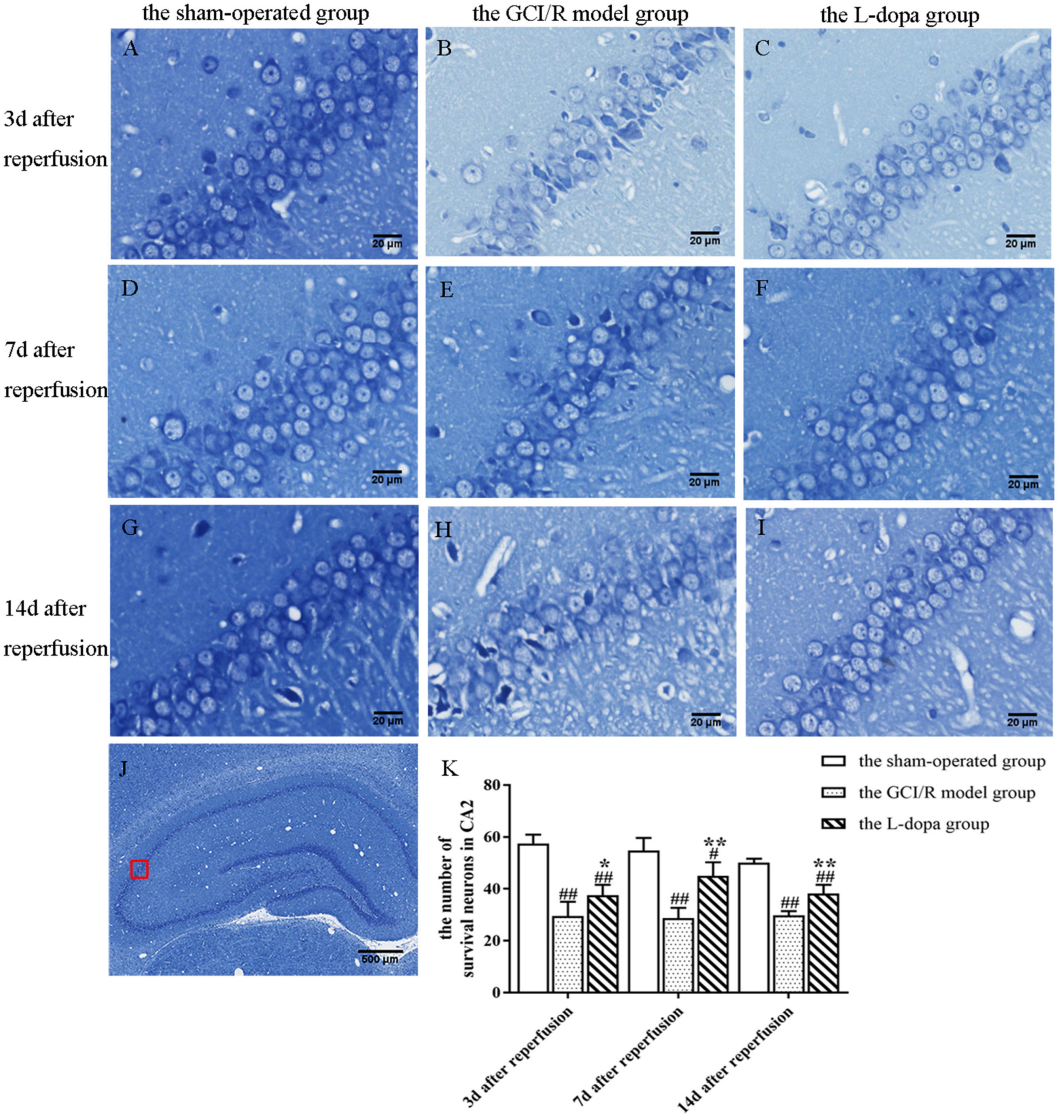
Nissl staining of hippocampal CA2 subfield. **(A–J)** Representative section for Nissl staining at 3, 7, and 14 days after GCI/R injury. Scale bar 500 μm for Magnification 4×, Scale bar 20 μm for Magnification 20×. **(K)** The mean number of survival neurons in the hippocampal CA2 at 3, 7, and 14 days after GCI/R injury. Data are shown as means ± SD. ##*p* < 0.01 vs. the sham-operated group, **p < 0.01 vs. the GCI/R model group. #*p* < 0.05 vs. the sham-operated group, *p < 0.05 vs. the GCI/R model group.

The hippocampal CA2 region was selected under a 20x objective lens for neuron counting. The sham-operated group, model group, and levodopa administration group were counted at the same time. The result was analyzed by single factor analysis of variance. The result showed that 3, 7, and 14 days after reperfusion, neuron counts in the hippocampus of the three groups all showed homogeneity of variance (*p* > 0.05), and the LSD test was used as a *post-hoc* test. There were statistical differences between the three groups [*F*_(2, 9)_ = 42.789, *p* < 0.001; *F*_(2, 9)_ = 31.132, *p* < 0.001; *F*_(2, 9)_ = 75.197, *p* < 0.001], the number of normal neurons in the global ischemia/reperfusion injury model group and the levodopa administration group were significantly smaller than that in the sham-operated group (*p* < 0.01). Compared with the model group, the number of neurons in the drug administration group increased significantly (*p* < 0.01) (Figure 4).

### Levodopa Reduced the Delayed Neuronal Death of Rat Hippocampus After the GCI/R Injury

**Figure 5:**
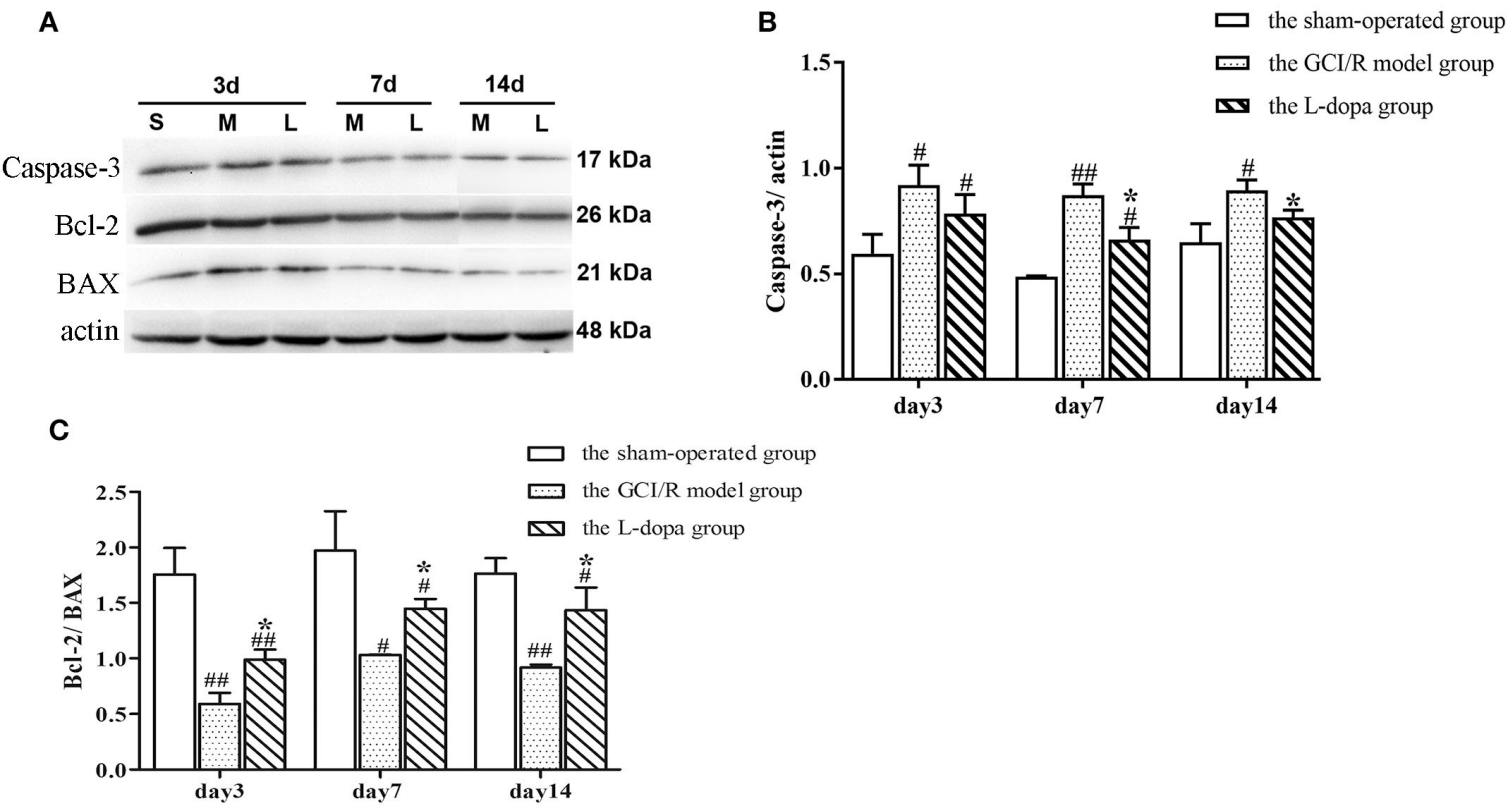
Western blot analysis of the expression of Caspase 3, Bcl 2, BAX proteins in hippocampal tissues. **(A)** The expression of Caspase 3, Bcl 2, BAX proteins in hippocampus. **(B)** The expression of Caspase 3 in the hippocampus at 3, 7, and 14 days after GCI/R injury. **(C)** The ratio of Bcl 2/Bax in the hippocampus at 3, 7, and 14 days after GCI/R injury. Data are shown as means ±SD. ##*p* < 0.01 vs. the sham operated group. #*p* < 0.05 vs. the sham operated group, *p < 0.05 vs. the G CI/R model group.

One-way analysis of variance was used to analyze the ratios of Caspase-3 and Bcl-2/Bax protein expression in the rat hippocampus of the sham-operated group, model group and drug administration group at 3, 7, and 14 days after reperfusion (Figure 5). The data of Caspase-3 protein expression in the rat hippocampus of the sham-operated group, model group and administration group at 3, 7, and 14 days of reperfusion all showed homogeneity of variance (*p* > 0.05), and the LSD test was used as a *post-hoc* test. The expression levels of Caspase3 in the rat hippocampus of the three groups at three time points were statistically different [*F*_(2, 6)_ = 9.571, *p* < 0.05; *F*_(2, 6)_ = 54.494, *p* < 0.01; *F*_(2, 6)_ = 12.007, *p* < 0.01]. The expression levels of Caspase3 protein of the model group was higher than that of the sham-operated group, and the difference was statistically significant (*p* < 0.05). The expression level of Caspase3 protein of the drug administration group was lower than that of the model group, and difference was statistically significant (*p* < 0.05) (Figure 5B).

The data of Bcl2/Bax ratio in the rat hippocampus of the sham-operated group, model group and drug administration group at 3, 7, and 14 days after reperfusion showed homogeneity of variance (*p* > 0.05), and the LSD test was used as a *post-hoc* test. The results of *post-hoc* test showed there was significant differences between the three groups in the Bcl2/Bax ratio in rat hippocampus at 3, 7, and 14 days after reperfusion [*F*_(2, 6)_ = 41.772, *p* < 0.001; *F*_(2, 6)_ = 14.888, *p* < 0.01; *F*_(2, 6)_ = 26.534, *p* < 0.01]. The Bcl2/Bax ratio in the rat hippocampus of the model group was lower than that of the sham-operated group (*p* < 0.05). The Bcl2/Bax ratio in the rat hippocampus of the drug administration group was significantly higher than that of the model group (*p* < 0.05) (Figure 5C).

### Levodopa Increased the Level of Dopamine in the Rat Hippocampus After GCI/R Injury

**Figure 6:**
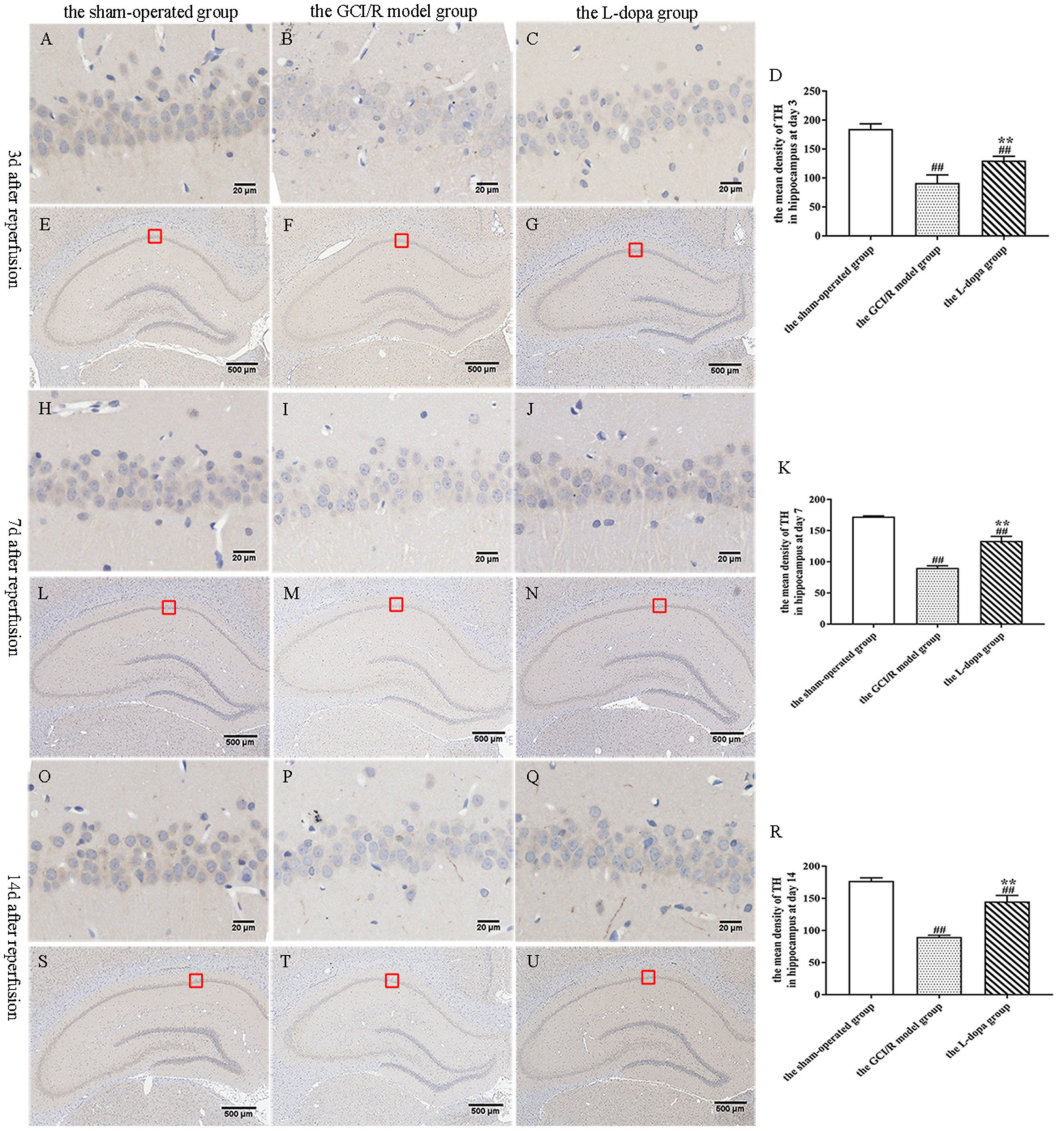
TH immunohistochemical staining of hippocampus. **(A–U)** Representative section for TH immunohistochemical staining at 3, 7, and 14 days after GCI/R injury. Scale bar 500 μm for Magnification 4×, Scale bar 20 μm for Magnification 20×. **(D)** The mean density of TH in hippocampus at 3 day after GCI/R injury. **(K)** The mean density of TH in hippocampus at day 7 after GCI/R injury. **(R)** The mean density of TH in hippocampus at day 14 after GCI/R injury. Data are shown as means ± SD. ##*p* < 0.01 vs. the sham-operated group, **p < 0.01 vs. the GCI/R model group.

TH is a rate-limiting enzyme for dopamine synthesis. This study detected the TH content in brain tissue sections, which can indirectly reflect the content of dopamine in neurons (Figure 6). One-way analysis of variance was used to analyze the mean optical density of TH-stained sections in the hippocampus of rats in the sham-operated group, the model group and levodopa administration group at the same time. The results of comparisons between the three groups at three time points showed homogeneity of variance (*p* > 0.05). One-way analysis of variance was used for statistical comparison, and the LSD test was used as a *post-hoc* test. The results showed that at different time points, the TH expressions in the rat hippocampus of the sham-operated group, the model group and drug administration group were statistically different [*F*_(2, 9)_ = 80.217, *p* < 0.001; *F*_(2, 9)_ = 302.717, *p* < 0.001; *F*_(2, 9)_ = 196.7, *p* < 0.001] (Figures 6D,K,R). Compared with the sham-operated group, the average optical density values of TH in the hippocampus of the model group and the drug administration group decreased significantly (*p* < 0.001). The expression of TH in the hippocampus of the levodopa administration group was significantly higher than that of the model group (*p* < 0.001). The results showed that levodopa can up-regulate the optical density of TH-stained sections in the rat hippocampus after GCI/R injury.

### Levodopa Improved the Synaptic Plasticity in the Rat Hippocampus After GCI/R Injury

#### Analysis of Synapse Ultrastructure in the Rat Hippocampus

**Figure 7:**
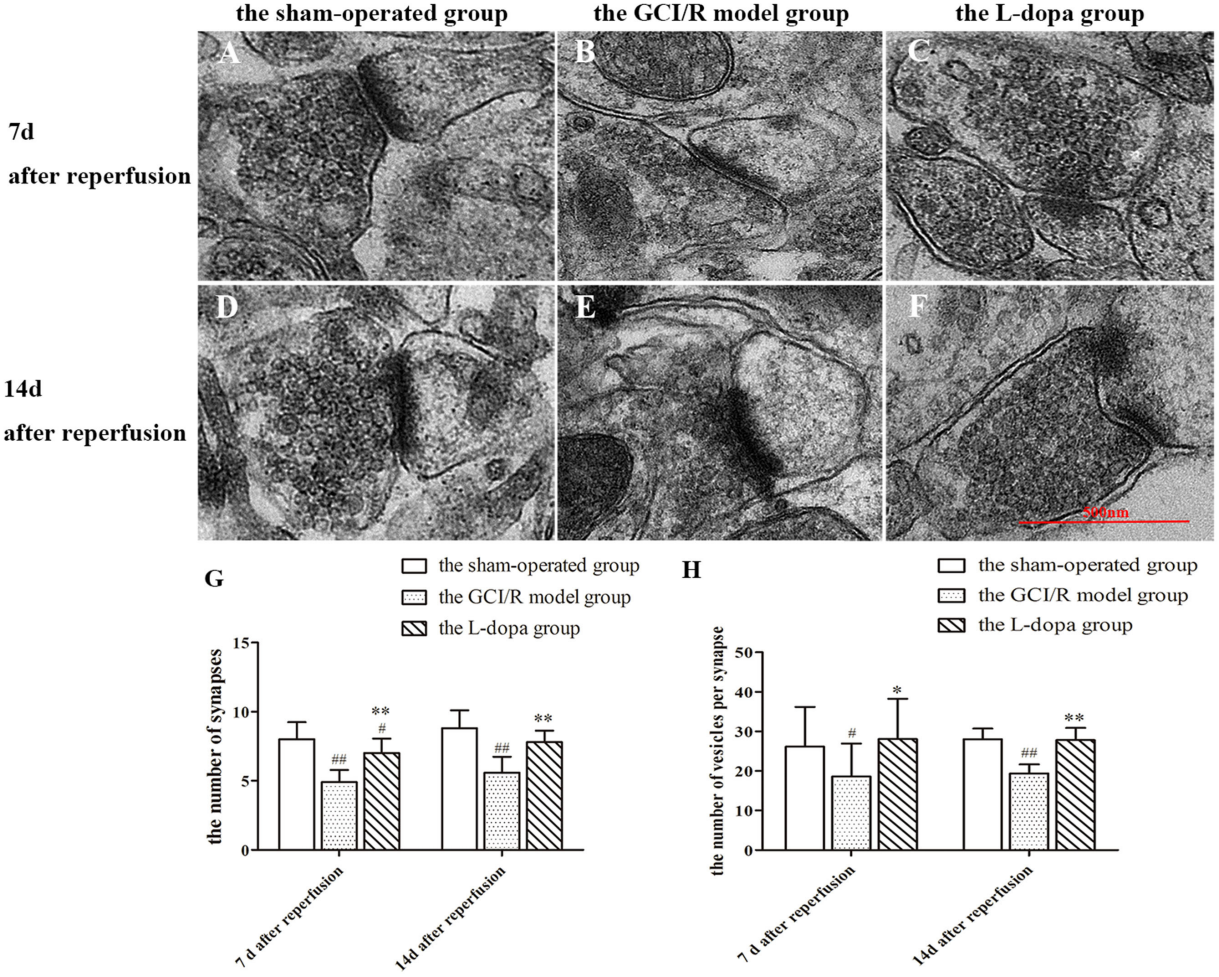
Ultrastructure of synapses in hippocampus. **(A–F)** Representative section for synaptic ultrastructure at 7 and 14 days after GCI/R injury. Scale bar 500 μm for Magnification 20,000×. **(G)** The number of synapses in hippocampal CA1. Magnification 6,000×. **(H)** The number of vesicles per synapse in hippocampal CA1. Data are shown as means ± SD. ##*p* < 0.01 vs. the sham-operated group, **p < 0.01 vs. the GCI/R model group. #*p* < 0.05 vs. the sham-operated group, *p < 0.05 vs. the GCI/R model group.

In this study, transmission electron microscopy was used to observe the ultrastructure of synapses in the hippocampal CA1 region at different time points in three groups, and the number of synapses was counted (Figure 7). In the sham-operated group, the front and rear boundaries of the synapse was clearly demarcated, the contours were complete, and there were many round and clear synaptic vesicles in the presynaptic terminal, which were densely and evenly distributed. In the model group, the presynaptic membrane in the hippocampal CA1 subregion was not clear, the synaptic vesicles were reduced, the membrane was split, and the synaptic space was blurred. In the levodopa administration group, the structure of synapse was closer to the control group, the boundary between the presynaptic membrane, and the synaptic space was clear, and the synaptic vesicles were densely distributed.

The numbers of synapses at different time points in the rat hippocampal CA1 subregion of the three groups were analyzed by using two-factor analysis of variance, all of which showed homogeneity of variance, there were differences between the three groups [*F*_(5, 39)_ = 14.123, *p* < 0.001], and the LSD test was used as a *post-hoc* test (Figure 7G). The *post-hoc* analysis showed that the number of synapses in the hippocampal CA1 subregion of the model group was significantly reduced compare with the sham-operated group (*p* < 0.01). The number of synapses in the drug administration group was significantly increased compared with the global cerebral ischemia/reperfusion in the model group (*p* < 0.01).

The numbers of synapses at the same time in the rat hippocampal CA1 subregion of the sham-operated group, the model group and levodopa administration group were analyzed by one-way ANOVA, all of which showed homogeneity of variance (*p* > 0.05), and the LSD test was used as a *post-hoc* test. The results showed that the numbers of synapses in the rat hippocampal CA1 subregion were significantly different at 7 and 14 days between the sham-operated group, the model group and the drug administration group [*F*_(2, 27)_ = 21.874, *p* < 0.001; *F*_(2, 12)_ = 16.062, *p* < 0.001]. Compared with the sham-operated group, the average optical density values of TH in the hippocampus of the model group and the drug administration group decreased significantly (*p* < 0.001). The expression of TH in the hippocampus of the levodopa administration group was significantly higher than that of the model group (*p* < 0.001). The results showed that levodopa can improve the structure and number of synapses in the rat hippocampal CA1 subregion after GCI/R injury.

#### Analysis of the Expression of Synaptic Plasticity-Related Proteins in Hippocampus

**Figure 8:**
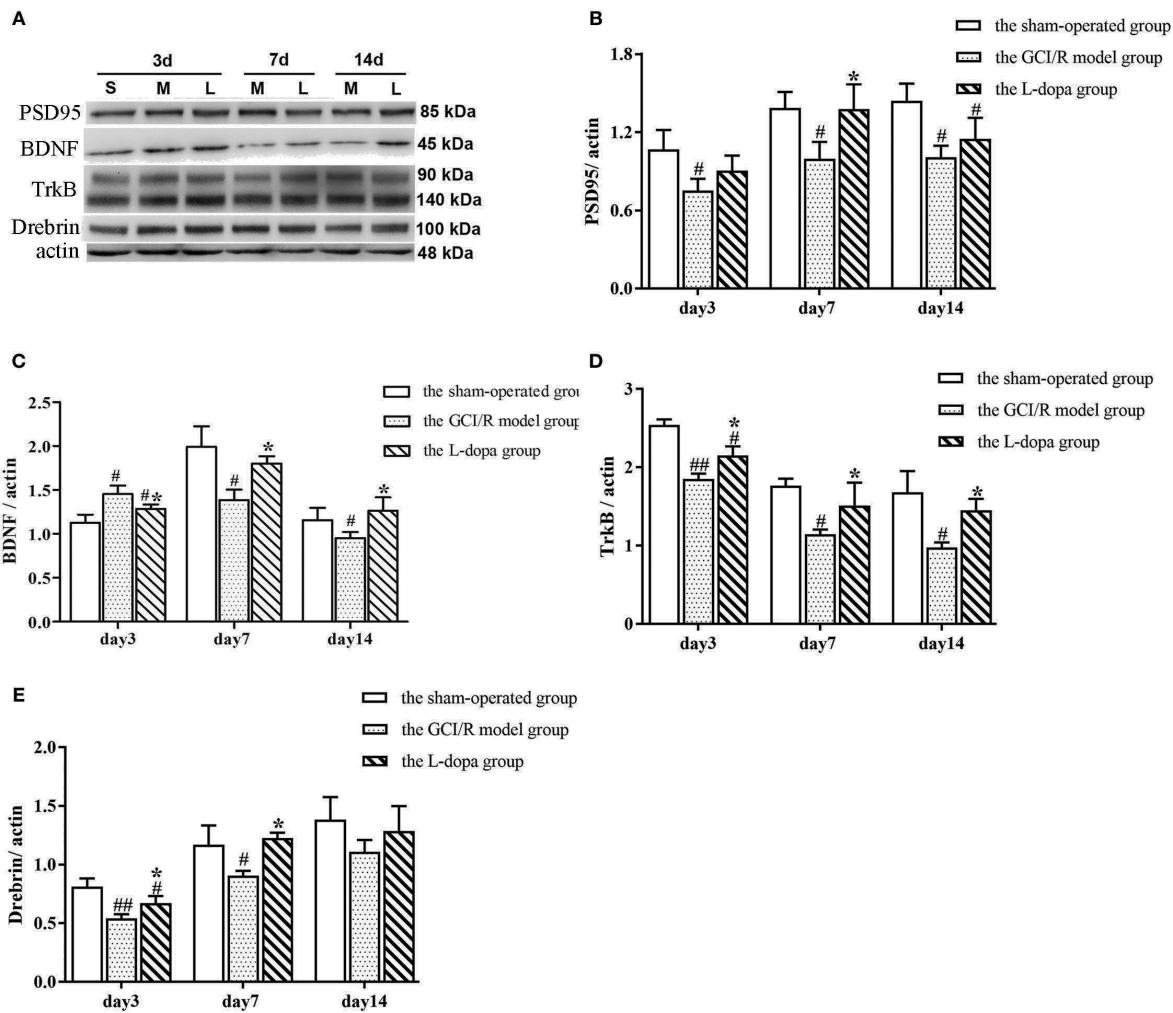
Western blot analysis of the expression of synaptic plasticity related proteins in hippocampal tissues. **(A)** The expression of PSD95, BD NF, TrkB, Drebrin proteins in hippocampus. **(B)** The expression of PSD95 **(B)**, BDNF **(C)**, TrkB **(D)**, and Drebrin **(E)** in the hippocampus at 3, 7, and 14 days after GCI/R injury. Data are shown as means ± SD. ##*p* < 0.01 vs. the sham operated group. #*p* < 0.05 vs. the sham operated group, *p < 0.0 5 vs. the GCI/R model group.

The data of PSD95 protein expression in the hippocampus of rats in the sham-operated group, model group, and drug administration group at 3, 7, and 14 days after reperfusion all showed homogeneity of variance (*p* > 0.05), which were analyzed using one-way analysis of variance, and the LSD test was used as a *post-hoc* test. The results showed there were significant differences in the PSD95 protein expression in the rat hippocampus between the three groups at 3, 7, and 14 days after reperfusion [*F*_(2, 6)_ = 5.121, *p* = 0.05; *F*_(2, 6)_ = 6.568, *p* < 0.05; *F*_(2, 6)_ = 8.598, *p* < 0.05]. The PSD95 protein expression in the hippocampus of the model group was significantly lower than that of the sham-operated group (*p* < 0.05). At 7 days after reperfusion, the PSD95 protein expression in the hippocampus of the drug administration group was significantly higher than that of the model group (*p* < 0.05) (Figure 8B).

The data of BDNF protein expression in the hippocampus of rats in the sham-operated group, model group and drug administration group at 3, 7, and 14 days after reperfusion all showed homogeneity of variance (*p* > 0.05), which were analyzed using one-way analysis of variance, and the LSD test was used as a *post-hoc* test. The results showed there were significant differences in the BDNF protein expression in the rat hippocampus between the three groups at 3 and 7 days after reperfusion [*F*_(2, 6)_ = 11.504, *p* < 0.01; *F*_(2, 6)_ = 11.151, *p* < 0.05]. At 3 days after reperfusion, the BDNF protein expression in the hippocampus of the model group was higher than that of the sham-operated group (*p* < 0.01). At 7 days after reperfusion, the BDNF protein expression in the hippocampus of the model group was significantly lower than that of the sham-operated group (*p* < 0.01). At 7 and 14 days after reperfusion, the BDNF protein expression in the hippocampus of the drug administration group was significantly higher than that of the model group (*p* < 0.05) (Figure 8C).

The data of TrkB protein expression at different time points in the rat hippocampus of the sham-operated group, model group and drug administration group showed homogeneity of variance (*p* > 0.05), which were analyzed using one-way analysis of variance, and the LSD test was used as a *post-hoc* test. The results showed there were significant differences in the TrkB protein expression in the rat hippocampus between the three groups at different time points [*F*_(2, 6)_ = 46.664, *p* < 0.01; *F*_(2, 6)_ = 8.902, *p* < 0.05; *F*_(2, 6)_ = 11.665, *p* < 0.01]. The TrkB protein expression in the hippocampus of the model group was significantly lower than that of the sham-operated group (*p* < 0.05). The TrkB protein expression in the hippocampus of the drug administration group was significantly higher than that of the model group (*p* < 0.05) (Figure 8D).

The data of Drebrin protein expression in the hippocampus of rats in the sham-operated group, the model group, and drug administration group at 3, 7, and 14 days after reperfusion all showed homogeneity of variance (*p* > 0.05), which were analyzed using one-way analysis of variance, and the LSD test was used as a *post-hoc* test. The results showed there were significant differences in the Drebrin protein expression in the rat hippocampus between the three groups at 3 and 7 days after reperfusion [*F*_(2, 6)_ = 17.105, *p* < 0.01; *F*_(2, 6)_ = 8.526, *p* < 0.05]. At 14 days after reperfusion, there was no statistically significant difference in the Drebrin protein expression between the three groups [*F*_(2, 6)_ = 1.882, *p* > 0.05]. At 3 and 7 days after reperfusion, the Drebrin protein expression in the hippocampus of the model group was significantly lower than that of the sham-operated group (*p* < 0.05), and the Drebrin protein expression in the hippocampus of the drug administration group was significantly higher than that of the model group (*p* < 0.05) (Figure 8E).

## Discussion

Global cerebral ischemia/reperfusion causes delayed death of neurons in the hippocampus, unbalanced dopamine content, and decreased synaptic density, which eventually leads to learning and memory disorders. The results of this study show that after levodopa administration, the neurological function and the learning and memory functions of rats with GCI/R injury were improved, the white matter integrity and gray matter density in the hippocampus were improved, the delayed death of neurons in the hippocampus was decreased, and the number of synapses and the expression of neuronal plasticity-related proteins were increased. Levodopa can repair the impaired learning and memory functions after GCI/R injury by improving synaptic plasticity in the rat hippocampus.

After cerebral ischemia/reperfusion occurs, there is secondary failure of cellular energy metabolism. The ATP in the brain is reduced and the function of the glutamate transporter is inhibited, which results in the accumulation of glutamate in the synaptic gap, cause excitotoxicity and excessive activation of the post-synaptic glutamate receptor, N-methyl-D-aspartate receptor (NMDAR), induce calcium influx and abnormal mitochondrial function, release more free radicals, and initiate apoptosis, which leads to delayed cell death (Jakaria et al., 2018). At the same time, the expression levels of post-synaptic density protein of 95 kDa (PSD-95) and BDNF-TrkB decrease, and the loss of hippocampal synapses and dendritic spines causes significant changes in the morphology of neurons and their synapses (Jakaria et al., 2018).

GCI/R injury causes not only delayed death of neurons but also delayed destruction of the dopamine system and imbalance of the dopamine content in the hippocampus. The change of dopamine content after cerebral ischemia/reperfusion injury was observed by using microdialysis technology. It was found that the dopamine content in the brain decreased rapidly after reperfusion for 80–120 min (Li et al., 2010). Li Bing et al. found that the content of dopamine neurotransmitters in the hippocampus increased significantly 6 h after GCI/R injury, which was 336.1% of the control group's dopamine content, but the dopamine content in the hippocampus started to decline 1 day after GCI/R injury, and, 3 days later, the dopamine content in the hippocampus was significantly lower than that of the control group (Li Bing et al., 2006).

Tyrosine hydroxylase is a catalytic enzyme and rate-limiting enzyme for dopamine synthesis. The content of tyrosine hydroxylase in neurons can indirectly reflect the level of dopamine in neurons. The result of tyrosine hydroxylase in this study is consistent with those of other studies, that is, the dopamine level in the hippocampus decreased and was lower than the normal level after cerebral ischemia/reperfusion injury. After intervention with levodopa, the level of dopamine in neurons increased, which indicated that levodopa could reduce the decrease of dopamine in the hippocampus after cerebral ischemia/reperfusion to a certain extent. Enhancing the activity of tyrosine hydroxylase and increasing the synthesis of endogenous dopamine can effectively improve the nerve function of rats after cerebral ischemia/reperfusion injury (Zhong et al., 2019). Obi et al. found that activation of the dopaminergic nervous system contributes to functional recovery in the chronic phase of stroke (Obi et al., 2018). Scheidtmann et al. also pointed out that levodopa combined with physical therapy is beneficial to the recovery of neurological function in patients with stroke (Scheidtmann et al., 2001). Earlier studies of our research group also showed that patients with persistent coma after resuscitation could have significant improvement in their disorders of consciousness after using levodopa or piribedil (Lixu Liu and Shi, 2006).

After cerebral ischemia, there will be structural changes in the relevant brain area, such as the increase or decrease of gray matter density and volume. It was reported that the VBM assessment of stroke patients showed a significant decrease in the gray matter volume of ipsilateral pre-central gyrus, paracentral gyrus, and contralateral cerebellum lobule VII and a significant increase in the gray matter volume of contralateral orbital frontal cortex and inferior frontal gyrus at the chronic stage of stroke (Cai et al., 2016). Drobyshevsky et al. found, in animal models of pre-natal ischemia and hypoxia, that microstructural changes occurred in multiple brain regions, FA decreased, and white matter volume decreased (Drobyshevsky, 2017).

BDNF is involved in the structural regulation of synapse production, maintenance, enlargement, and modification, as well as in the functional regulation of neurotransmission and receptor activity, and promotes the growth of dendrites and axons (Chen et al., 2018). Dopamine can increase the expression of BDNF in the hippocampus by activating dopamine receptors (Berton et al., 2006; Hasbi et al., 2011), thereby improving learning and memory functions (Kowianski et al., 2018). TrkB is a receptor with a high affinity for BDNF, which is expressed in both the pre-synapse and post-synapse. BDNF-TrkB can increase the plasticity of hippocampal neurons by regulating the expression of PSD95 and Drebrin as well as regulating the learning and memory at the cellular level (Li et al., 2018). Studies on DTI showed that the content of BDNF is related to the FA value in the hippocampus; the lower the BDNF content, the lower the FA value and the worse the integrity of white matter in the hippocampus (Chen et al., 2016). The results of this study showed that the administration of levodopa after GCI/R injury increased the number of synapses, and the density of vesicles in the hippocampus, while also increasing the expression of PSD95 and Drebrin, promoted the repair of white matter and gray matter of the hippocampus and enhanced the synaptic plasticity. This may be related to the increase of BDNF and TrkB protein expression in the rat hippocampus after levodopa administration.

This study has certain limitations, and only detected the effect of levodopa on synaptic plasticity in the hippocampus after GCI/R injury. The ventral tegmental area can project dopamine not only to the hippocampus but also to the pre-frontal lobe (Tuesta and Zhang, 2014), and the pre-frontal-hippocampal circuit participates in the learning and memory process (Shin et al., 2019). Injury of the pre-frontal-hippocampal circuit results in impaired functional integrity of nerve conduction pathways, which leads to working memory impairment in patients consequently (Lundeberg et al., 2007). In future studies, we will continue to explore the effect of levodopa on the pre-frontal-hippocampal circuit after GCI/R injury.

To conclude, our study shows levodopa can improve the learning and memory functions after GCI/R injury occurs, by enhancing the synaptic plasticity in the hippocampus. Levodopa is the most commonly used medicine in the therapy of Parkinson's disease, and its safety has been proven. A new approach was proposed in this study for brain functional rehabilitation therapy after cardiopulmonary resuscitation.

## Data Availability Statement

The raw data supporting the conclusions of this article will be made available by the authors, without undue reservation.

## Ethics Statement

The animal study was reviewed and approved by the ethics committee of Capital Medical University, and the ethical number is: AEEI-2018-096.

## Author Contributions

WW and XL carried out the experiment and wrote the manuscript with support from HS and ZY. LL conceived the original idea. TZ and YY supervised the project. All authors contributed to the article and approved the submitted version.

## Conflict of Interest

The authors declare that the research was conducted in the absence of any commercial or financial relationships that could be construed as a potential conflict of interest.

## Abbreviations

ANOVA: analysis of variance
H&E: hematoxylin and eosin
mNSS: modified Neurological Severity Score
SD: Sprague-Dawley.
